# Filipino Child Health in the United States: Do Health and Health Care Disparities Exist?

**Published:** 2007-03-15

**Authors:** Joyce R Javier, Lynne C Huffman, Fernando S Mendoza

**Affiliations:** Division of General Pediatrics, Stanford University School of Medicine; Division of General Pediatrics, Stanford University School of Medicine, Palo Alto, Ca; Division of General Pediatrics, Stanford University School of Medicine, Palo Alto, Ca

## Abstract

**Introduction:**

Filipinos are the second largest Asian subgroup in the United States, but few studies have examined health and health care disparities in Filipino children. The objectives of this review are 1) to appraise current knowledge of Filipino children's health and health care and 2) to present the implications of these findings for research, clinical care, and policy.

**Methods:**

We identified articles for review primarily via a Medline search emphasizing the terms *Filipino* and *United States* crossed with specific topics in child and adolescent health that fall under one of Healthy People 2010's 28 focus areas.

**Results:**

Filipino children are underrepresented in medical research. Studies that compare Filipino children and adolescents with white children or children of other Asian Pacific Islander subgroups suggest disparities with regard to gestational diabetes, rates of neonatal mortality and low birth weight, malnutrition in young children, overweight, physical inactivity and fitness, tuberculosis, dental caries, and substance abuse. Studies that compare Filipino adults with white adults describe adult Filipino health problems similar to those of Filipino children, including higher rates of diabetes, hypertension, and metabolic syndrome. Health care disparities remain to be determined.

**Conclusion:**

Health and health care disparities appear to exist for Filipino children, but more research is needed to confirm these findings. Practitioners serving this population need to consider social and cultural factors that can increase or diminish risk for health problems. There are priorities in research and policy that, if pursued, may improve the health care and health outcomes of Filipino children.

## Introduction

The population of Asian Pacific Islander (API) children in the United States is expected to more than double by 2025 (1). Yet, we have only a limited understanding of the health and health care issues that characterize these children. Many studies that include API children report aggregated results, which mask key variations in health status among API subgroups. Moreover, many recent national surveys of children and adolescents do not collect data on API subgroups (2,3).

**Figure 1:**
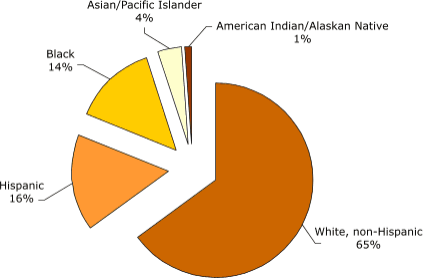
Percentage of U.S. children (Aged 0–18) by racial or ethnic group, 2000. All categories include those of mixed race or mixed ethnicity (7). Calculated by Joyce R. Javier from U.S. Census Bureau, Census 2000 Summary File 2 (SF 2) 100-Percent Data files.

**Figure 2:**
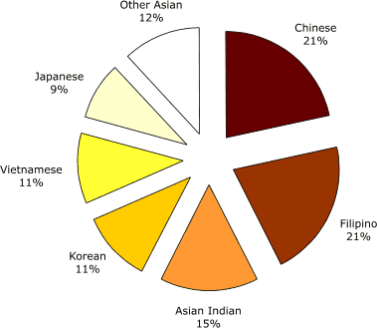
U.S. Asian children (Aged 0–18) by subgroup, 2000. All categories include those of mixed race or mixed ethnicity (7). Calculated by Joyce R. Javier from U.S. Census Bureau, Census 2000 Summary File 2 (SF 2) 100-Percent Data files.

Filipinos are the second largest API subpopulation in the United States (4). On average, one in five Asians in the United States is Filipino (5). Most Filipinos live in California or Hawaii; however, Filipino populations in other states have more than doubled in the past decade (e.g., Texas, Florida). California is home to the largest Filipino population with more than 1 million, and Hawaii ranks second. Between 1990 and 2000 the U.S. Filipino population grew by 68.1% (6). Figures 1 and 2 display the distributions of the U.S. child population by ethnicity and by Asian population subgroup (7), respectively.

**Table:** 

**Table:** 

Studies that compare Filipino adults with other API or white adults have found disparities in areas such as cancer, cardiovascular health, and mental health (8,9). Little has been published about the health of Filipino children and adolescents. In this descriptive review of the literature, we compare the health of Filipino and white children to determine whether Filipino children are among those children in the United States that experience health and health care disparities. When data are available, we highlight differences between Filipino and other Asian subgroups. We also present important health and health care issues for which data are available on Asians but not on Filipinos specifically.

## Methods

We identified articles for review primarily via a Medline search of the terms *Filipino* and *United States* crossed with specific topics in child and adolescent health that fall under one of Healthy People 2010's 28 focus areas. Studies selected for review were for the most part limited to those that were published between 1985 and 2005, were written in English, had subjects in the United States, compared Filipinos or Asians with other racial or ethnic groups, and addressed specific topics in child and adolescent health. Topics addressed were access to quality health services, maternal and infant health, nutrition, oral health, overweight, physical activity and fitness, respiratory diseases, infectious diseases and immunizations, sexually transmitted diseases, substance abuse, tobacco use, injury and violence prevention, mental health, and conditions highly prevalent among adults on which there are no studies with Filipino children as subjects (e.g., diabetes, heart disease).  These topics were selected because they fall under one of Healthy People 2010's 28 focus areas (10). We further examined the references listed in the identified articles and, where available, followed electronic links to related articles through the PubMed search engine.

In this article the term Filipino denotes any U.S. resident originating from any of the original peoples of the Philippine Islands. We do not use the terms API and Asian interchangeably. API refers to Asians and Pacific Islanders together as one group. When we refer to these two groups separately, the term Asian does not include Pacific Islanders. An Asian is a person whose origins are in any of the original people of the Far East, Southeast Asia, or the Indian subcontinent, including the Philippine Islands. A Pacific Islander** **is a person whose origins are in any of the original peoples of Hawaii, Guam, Samoa, or other Pacific Islands. Pacific Islanders are a separate category from Asians, although they are sometimes included with Asians or relegated to an *other* category.

## Results

Health disparities can manifest in two major areas: health and health care. In this review, we first address health care, specifically examining access to quality health care. We then focus on the health of Filipino youth in three categories: infant and young child health, school-age youth and adolescent health, and relevant adult health problems. We present the identified health studies with specific data on Filipino infants and young children (Table 1), school-age youth and adolescents (Table 2), and relevant adult diseases (Table 3).

### Access to quality health services

Research is lacking on the quality of medical care among Filipino children. With respect to health care access, national data show that Filipino children in the United States are more likely than white children to have health insurance. Filipino youths also have more favorable health status than white youths as indicated by less frequent school absence, less learning disability, less use of prescription medications, and fewer chronic conditions. However, Filipino children are more likely than white children to be without a usual place for receiving health care and not to have had contact with a health professional within the previous 12 months (11). Also, compared with Filipino adolescents living in the United States for short periods, Filipino adolescents living in the United States for long periods tended to report more physical health problems (12). These studies suggest that Filipino children face barriers to health care access and use that extend beyond health insurance issues, highlighting the need to consider generational status (i.e., whether the  child is a first, second, or third generation immigrant) and time spent in the United States when considering risk for health problems.

### Infants' and young children's health (aged 0–5 years)

#### Maternal and infant health

Studies have documented mixed perinatal outcomes for Filipinos, and when these outcomes are worse compared to whites, familiar risk factors (e.g., lack of prenatal care, smoking status) do not completely explain this variation. Compared with white mothers, Filipino mothers have similar infant, neonatal, and postneonatal mortality rates (13). However, Filipino women are at increased risk for delivering infants who are of moderately low (defined as 1,500–2,499g) and very low birth weight (defined as 500–1,499g) (14), preterm (15), or stillborn (16).

For Filipino women, the association between maternal place of birth and child health has been mixed. National data demonstrate a pattern consistent with the epidemiological paradox in that, compared with the offspring of U.S.-born mothers, the offspring of Philippine-born mothers have more favorable birth weights as well as lower infant mortality rates (13,17,18). Philippine-born mothers are also less likely to use alcohol and tobacco and have inadequate prenatal care during their pregnancies but are more likely to have inadequate weight gain (less than 22 pounds) (18). A study in Hawaii found that, compared with U.S.-born Filipinos, foreign-born Filipinos had a higher risk of preterm delivery and low birth weight (19).

According to the National Vital Statistics Report (17), in 2002 Filipino mothers (data not available by place of birth) had the highest rate of gestational diabetes among all measured subgroups at 59.8 per 1,000.  Another study using national data reported that Philippine-born Filipino mothers are significantly more likely to have diabetes during pregnancy than U.S.-born Filipino mothers (20). The impact of the mother's place of birth on diabetes prevalence may be partially explained by the older childbearing age of Filipino immigrant mothers, but differences in diabetes prevalence remain even after adjusting for differences in maternal age and other sociodemographic characteristics.

Weitz et al examined teen pregnancy among API subgroups and reported that when data on APIs are disaggregated, Filipinos have the highest percentage of births to teens (6%) among California's six largest API groups (Chinese, Filipino, Vietnamese, Korean, Indian, and Japanese) (21). Compared with white teens, Filipino teens are more likely to request a pregnancy test and no other services from a provider, suggesting that they are sexually active but not looking for birth control. According to national data, U.S.-born Filipino mothers are more likely to be under age 20 than foreign-born Filipino mothers (11.0% vs 2.7%) (17). These studies reveal the importance of considering moderating variables (e.g., maternal place of birth, regional variations, maternal age, and genetic predisposition) when examining maternal and infant health outcomes in Filipinos.

#### Nutrition

There have been a limited number of nutrition studies on Filipino children because they are not sampled as a group in national or large regional studies. A longitudinal study of immigrant school-aged children in San Francisco revealed that all immigrant children, including those from the Philippines, showed catch-up growth in weight and height. This finding suggests that they arrived in this country with a deficiency in either height or weight and that they were malnourished before arriving in the United States (22). Another study of children participating in Hawaii's Women, Infants, and Children program found that, among 1-year-olds, Filipinos had the highest percentage of underweight (30.2%); among children aged 2 to 4 years, Filipinos had the highest percentage of short stature (19.0%) (23). These two studies suggest that Filipino immigrant children may be affected by a statistically significant degree of relative malnutrition.

#### Oral health

There are no national data on the prevalence of dental caries among Filipino children. In Hawaii, Filipino children stand out as having one of the highest rates of dental caries (defined as decayed and filled teeth), a rate nearly three times the national average. The proportion of Filipino children with baby bottle tooth decay and unmet dental treatment needs is higher than the proportion of white children (24). A study using community focus groups in the Northern Mariana Islands, a commonwealth of the United States, found that the low value of baby teeth and the negative treatment experiences parents had with painful dental care were major determinants of health beliefs surrounding oral health care for their children (25). Qualitative research allows for cultural tailoring of interventions, which may make dental treatment more acceptable to the targeted group. For example, our literature review revealed that advertising claiming that preventive visits are pain- and fear-free, in contrast to the experiences of parents when they were young, could be an important strategy to promote dental visits earlier in childhood.

### School-age youth and adolescent health (aged 6–18 years)

#### Overweight, physical activity, and fitness

A study of fifth, seventh, and ninth graders in California demonstrated that Filipino males were more likely to be obese (defined as Body Mass Index [BMI] ≥85th percentile) than white males, and Filipino females were more likely to be obese than white females (26). In a study that did not separate males from females, Filipino adolescents had a lower prevalence of obesity than white adolescents. However, Filipino adolescents born in the United States were more than twice as likely to be obese as were those born outside the United States (27).

Research on perceptions of obesity and body- or self-dissatisfaction reported varied results based on age and sex. A study of college students found that Filipino males had the highest BMI and were the most body- or self-dissatisfied of all male comparison groups (28). A study of third grade children in 13 Northern California public elementary schools revealed that Filipino girls were as likely to have overweight concerns and body-dissatisfaction as white girls (29).

Filipino adolescents were significantly less likely to be physically active than white adolescents (30). Filipino females (aged 10–15 years) and Filipino males (aged 10–11 years) also exhibited slower mile run or walk times than white boys and girls (26). The foregoing studies suggest that estimates of the prevalence of obesity, physical activity, and body dissatisfaction among Filipino children vary according to age and immigration status.

#### Respiratory diseases

There are no national prevalence data available on Filipino children for asthma, a highly prevalent chronic condition. According to a California survey of 7th, 9th, and 11th grade students conducted from 2001 to 2003, Filipinos have the highest lifetime asthma prevalence rates (23.3%) among API subgroups (31). This prevalence rate is lower than the rate for African Americans, higher than the rate for whites and Asians overall, and comparable with the rate for Puerto Ricans, an already recognized high-risk population.

#### Infectious diseases and immunizations

Immigrant children from the Philippines are the second largest immigrant group in the United States with tuberculosis (32). A study of targeted school-based tuberculosis screening among high-risk adolescent populations in San Diego revealed that Filipinos are more likely to have positive tuberculin skin test (TST) results than whites. Although the Bacille Calmett-Guerin (BCG) vaccine was mentioned as a potential source of false-positive TST results, researchers were unable to correlate BCG vaccination with size of skin test induration because of lack of reliable data on past BCG vaccinations (33).

We were unable to find any studies reporting national or state immunization rates for Filipino children. Perinatal transmission is the most common mode of hepatitis B transmission, and the remainder of hepatitis B carriers are usually infected during early childhood. Among pregnant Asian American women, the prevalence of hepatitis B surface antigen positivity is higher among women born in the Philippines (5.1%) than among Asian American women born in the United States (2.0%) (34). A study in Los Angeles County found that about one third of Filipino fourth-grade students had their full series of hepatitis B vaccine (35). Both studies reveal the need to raise awareness that hepatitis B virus plays a role in the etiology of liver carcinoma and that infection is largely preventable through hepatitis B vaccination.

#### Sexually transmitted diseases

Filipino adolescents appear to be at high risk for contracting sexually transmitted diseases. In San Francisco, they have sexual activity rates similar to those of white students and higher than those of Chinese students (36,37). Filipino adolescents are less likely to have knowledge of HIV prevention, to talk with their parents about sexual issues, or to use contraception once sexually active (21,37,38).  Furthermore, a study of Filipino adolescents in Los Angeles revealed that knowledge of HIV prevention was not associated with condom use at last intercourse. Rather, high self-efficacy with respect to condom use (i.e., confidence in one's ability to put a condom correctly on oneself or partner) and carrying condoms was associated with high condom use at last intercourse (39). These findings suggest that traditional variables related to AIDS risk behavior among non-Asian populations may not explain condom use among Filipinos. Future studies should identify determinants of AIDS risk behavior among Filipinos as a first step toward developing culturally relevant AIDS prevention programs for these groups. For example, research examining communication about sex in Filipino families revealed that respect for parental authority directly conflicts with open discussions with children that are promoted in U.S. culture. Recognizing generational differences in acculturation that may lead to impaired transmission of values may be important in developing interventions addressing adolescent sexual health (38).

#### Tobacco use and substance abuse

Filipino adolescents have prevalence rates similar to those of whites for inhalant, tobacco, and alcohol use (40). Multiple studies that include API subgroup analyses show that Filipinos use alcohol, tobacco, and drugs at rates that are much higher than previously reported from surveys that aggregate APIs and thus probably have an unrecognized need for treatment (41,42).

Substance abuse in Filipino youth also appears to be related to mental health problems. A study in San Francisco found that Filipinos attribute taking drugs to feeling isolated and depressed shortly after immigrating to the United States and indicate that gang members initiate other youths into drug use (42). Among Filipino adolescent females in California, cigarette smoking is associated with depression and low self-esteem. In contrast, alcohol use is associated with high self-esteem in Filipino males (43).

#### Injury and violence prevention

A study in Hawaii revealed that Filipino parents have had to alter their child management style in the United States. Although they are less likely to exhibit verbally and physically aggressive behavior toward their children than Polynesian American parents, Filipino parents are faced with the need for more direct parental involvement than was customary in the Philippines, where the extended family shared in the training and discipline of children (44).

#### Mental health

Several studies conducted with small samples of Filipinos show differences in self-esteem and depression scale scores based on sex and immigration status. In Hawaii, Filipino females have higher mean depression scores than Filipino males, as measured by the Center for Epidemiologic Studies Depression Scale (45). Research from the Children of Immigrants Longitudinal Study reveal that low self-esteem was associated with being Filipino (46). Similar findings were supported in a separate qualitative study in two California cities (47). This study found that when asked what it means to be Filipino, the most prevalent response consisted of statements about family as the center. The family can offer a positive base of Filipino identity for Filipino youth; however, it also is a source of stress and alienation that has been associated with depression and suicidal thoughts. Interviews in high schools revealed that counselors were "concerned about the mental health of Filipino students as a group" because they are averse to seeking counseling for fear that their parents will find out (47).

### Relevant diseases in Filipino adults

It is imperative to consider adult diseases among Filipinos so that preventive measures can be started in childhood. Given the relatively limited data on Filipino children's health, examining diseases of Filipino adults provides some insight regarding the prevalence of certain conditions that may be higher among Filipino children than children of other races. For example, Filipino adults have a higher prevalence of diabetes and hypertension than whites and other API subgroups (48-51).

The use of BMI to define overweight and obesity across populations has been questioned in numerous studies because the relationship between BMI and risk for diabetes, hypertension, and metabolic syndrome in the Filipino adult populations differs from that in white populations (52,53). For example, compared with white women, Filipino women have a higher prevalence of diabetes and metabolic syndrome despite the fact that 90% of Filipino women were not defined as obese (54). This study suggests that the high prevalence of diabetes in Filipinos may be missed by health care providers because they are not obese by Western standards.

## Discussion

Our literature review yielded two important findings. First, it identified disparities in Filipino child health and health care, a critical first step because considering social and cultural factors that influence health and health care can help us to begin to understand these disparities. Some factors may be protective in terms of health outcomes or they may increase risk. Second, we identified areas of Filipino child health in need of more research.

### Filipino children and adolescents: health disparities do exist and health care disparities remain to be determined

Our literature review suggests that disparities in Filipino child and adolescent health do exist and are notable with regard to gestational diabetes, neonatal mortality and low birth weight, malnutrition, overweight, physical inactivity, tuberculosis, dental caries, and substance abuse. In addition, there is a high prevalence of diabetes, hypertension, and metabolic syndrome among Filipino adults, which is significant because of growing evidence that these diseases are affected by events during gestation and early childhood (55,56) and by health-related behavior that is established in childhood and adolescence, such as eating preferences, exercise patterns, and tobacco use (56).

### Factors that may contribute to health disparities for Filipino children

Social and cultural factors appear to play an important role in the health of Filipino children. Fundamental values in Filipino culture include respect for authority figures*,* *pakikisama*, and *hiya* (57). In general, Filipino adolescents are less willing than white adolescents to openly disagree with their parents, and they place less emphasis on autonomy (58). *Pakikisama* (family unity and closeness) emphasizes smooth interpersonal relationships and reflects a high value on family, harmony, and conflict avoidance (57). Filipinos often focus on child-rearing practices that develop group-oriented behaviors in their children, whereas mainstream U.S. culture often emphasizes independent, individualistic, and self-reliant behaviors. *Hiya* is shame and is a motivating factor behind behavior. Filipino culture, like other Asian cultures, holds that a child's behavior is a reflection of family upbringing. This cultural perception may explain a delay in seeking services for stigmatized problems, such as teen pregnancy and mental illness (59-62).

Lack of communication and cultural understanding between Filipino youths and their parents may be a source of intergenerational conflict, especially during adolescence when Filipino youths may become more assertive because of their Western acculturation towards individuality (63). Intergenerational conflict may affect where a child or an adolescent receives help and support. In an unpublished survey of 254 Filipino youths aged 15 to 17 years in Vallejo, California, 65%–85% felt their parents would not be supportive if they were aware of an alcohol or drug problem or unwanted pregnancy. Also, 76% knew of no place in the Filipino community where they would feel safe seeking help with personal problems (47). This finding is of special concern given the empirical evidence that depression affects Filipino youths and that adolescent health risk behaviors increase with each generation of Filipino youth. These behaviors include early sexual activity, nonuse of birth control, delinquency, use of violence, and substance abuse (12).

The cultural and social factors presented are meant to increase awareness. However, given the heterogeneity of this group (i.e., variability in immigration history and demographics), it is important never to assume that every Filipino family adheres to particular values.

### Needed research

There are inadequate data to allow for any firm conclusions regarding disparities experienced by Filipino children and adolescents in the areas of health care access, use, and quality; injury and violence prevention; chronic conditions such as asthma; and mental illness. Filipinos and other API subgroups need to be included in research in these areas so that we can determine where disparities exist and monitor progress in addressing these disparities over time (2,64). In addition, the quality of data must be examined by developing valid and reliable research instruments for use with this population. Measures used with other immigrant groups can be modified. For example, a Short Acculturation Scale for Hispanics has been cross-culturally validated for use with Filipino adults, and the Center for Epidemiologic Studies Depression Scale and State-Trait Anxiety Inventory has been evaluated for use in Filipino adolescents (65-67). When collecting data, researchers need to know immigration status variables such as whether subjects are first or second generation immigrants and the time subjects have been in the United States (68).

Finally, interventions need to be designed to achieve equity in health and health care for Filipino youths. Whenever possible, the strengths of Filipino communities (i.e., emphasis on family and interpersonal relationships) should be used. Some interventions have been implemented. For example, the National Institutes of Health (NIH) National Heart, Lung, and Blood Institute funded a community-based partnership to investigate health practices related to cardiovascular disease in the Filipino community (69). Currently, the NIH National Center on Minority Health and Health Disparities is funding a project that is using community-based participatory research (i.e., research that encourages investigators and community members to collaboratively answer questions posed by the community, collect data locally, and communicate results directly to the community for its use [70]) to improve health care access and health status for cardiovascular disease for Filipino Americans with hypertension living in New York City (70). Similar community-based participatory research has been described in the area of teen pregnancy prevention in Filipino families (38,59).

### Clinical care implications

The social and clinical factors discussed here have significant implications for Filipino children who depend primarily on their parents to access and receive appropriate medical services. For example, health screening and education for stigmatized problems (e.g., tuberculosis) need to take cultural beliefs such as *hiya* into consideration (62).

For those who care for Filipino adolescents, discussing and assuring confidentiality may assist in getting these young people to open up about their problems. Both physical and mental health care practitioners can help parents develop communication skills for interacting with their often more acculturated children. Cross-cultural awareness can bridge the intergenerational and intercultural gaps between parents and children and facilitate the development of a bicultural identity (71). Increased ethnic identity and a greater appreciation of the struggles of immigrant families could improve intergenerational communication and lead to increased self-esteem and fewer risk behaviors among youths, a perspective with some empirical support (59,73,74).

### Policy implications

To address the Healthy People 2010 goals for APIs, health services research needs to include API subgroups. On the national level, APIs can be oversampled in national surveys such as the National Health and Nutrition Examination Survey. On the state and local level, increased funding should be made available for regions where there are substantial numbers of APIs (67). For example, the California Health Interview Survey conducted by the Center for Health Care Policy at the University of California, Los Angeles, is the first large-scale state survey to include API subgroups (75).

The need for cultural sensitivity and culturally effective care will only increase as health providers attempt to meet the needs of an increasingly racially diverse population (76,77). Proposed bills requiring cultural competency should be supported and such training should include information on Filipinos as well as other API subgroups.

### Conclusion

The year 2006 marked the centennial anniversary of the start of Filipino immigration to the United States. Despite their long history in the United States, Filipinos are understudied in medical research. This review reveals that Filipino children and adolescents are an important, yet hidden, minority group with multiple health needs. Since APIs are the fastest growing minority group within the United States, it is imperative that we address gaps in knowledge for all API subgroups. After understanding their health needs and recognizing their social and cultural strengths, we can then develop culturally appropriate interventions that work toward the goal of a healthier Filipino population with an improved quality of life.

## Figures and Tables

**Table 1:** Studies on Filipino Children's Access to Quality Health Services and on Filipino Infant and Young-Children's Health

**First Author**	**1. Data Source** **2. Sample Size^a^** **3. Methodology**	**Principal Findings**
**Yu (11)**	1. National Health Interview Survey, 1997-2000 2. 292 3. Cross-sectional study	Compared with white children, Filipino children were less likely to lack health insurance, miss school because of illness or injury, or have a learning disability and more likely to be without contact with a health professional within the past 12 months.
**Harris (12)**	1. National Longitudinal Study of Adolescent Health, 1995 2. 673 3. Cross-sectional study	Foreign-born Filipino adolescents had fewer health problems (e.g., learning difficulties, obesity, and asthma) than U.S.-born Filipino adolescents with foreign-born parents or whites. Filipino adolescents living in the United States for a long period tended to report more physical health problems than those here for a short time.
**Mathews (13)**	1. U.S. Linked Birth/Infant Death Data Set, 2002 2. 33,016 3. Descriptive analysis	Compared with white mothers, Filipino mothers had a similar infant mortality rate (5.7 vs 5.8 per 1000 births), neonatal mortality rate (4.1 vs 3.9 per 1000), and postneonatal mortality rate (1.7 vs 1.9 per 1000).
**Fuentes-Afflick (14)**	1. California birth certificates, 1992 2. 15,357 3. Cross-sectional study	Compared with white mothers, Filipino women were more likely to have both very low (OR, 1.38; 95% CI, 1.10-1.74) and moderately low birth weight infants (OR, 1.49; 95% CI, 1.35-1.64).
**Alexander (15)**	1. Hawaii Linked Birth/Infant Death Data Set, 1979-1989 2. 4521 3. Cross-sectional study	Pregnant Filipino women were more likely to be 35 years or older, less likely to initiate prenatal care in the first trimester, and more likely to have less than 12 years of education than white pregnant women. Among Filipinos, blacks, and whites, blacks were most likely to have preterm deliveries (11.09%), Filipinos next (10.17%), and whites least likely (6.45%). Risk for low birth weight was higher for Filipinos (OR 1.37; 95% CI, 1.20-1.57) and blacks (OR 1.91; 95% CI, 1.69-2.15) than for whites.
**Walton (16)**	1. Kaiser Permanente, Northern California medical records, 1995-1997 2. 1955^b^ 3. Cross-sectional study	The rate of stillbirth was substantially higher at nearly every serum chorionic gonadotropin concentration for blacks, Filipinos, Pacific Islanders, other, and unknown ethnic groups than for whites (range, 4-7 per 1000 for groups other than whites compared with 2 per 1000 for whites).
**Martin (17)**	1. U.S. birth certificates, 2002 2. 33,016 3. Descriptive analysis	Filipino mothers were as likely as white mothers to have prenatal care (85% vs 87.7%) and less likely to report tobacco use (2.9% vs 12.3%). Filipino infants were more likely to be preterm (12.7% vs 11.1%) or have a low birth weight (8.6% vs 6.8%) than white infants. Compared with foreign-born Filipino mothers, U.S.-born Filipino mothers were more likely to be under age 20 (11.0% vs 2.7%), report tobacco use (6.6% vs 1.8%), and have infants who were preterm (13.4% vs 12.5%) or low birth weight (9.5% vs 8.4%). Filipino women had the highest rate of gestational diabetes at 59.8 per 1000 births compared with whites (31.6), Blacks (30.4), American Indians (56.2), total APIs (54.0), Chinese (47.9), Japanese (42.5), Hawaiian (49.2), and other APIs (55.1).
**Landale (18)**	1. U.S. Linked Birth and Infant Death Data, 1989-1991 2. 68,505 3. Cross-sectional study	Compared with U.S.-born Filipino mothers, foreign-born Filipino mothers were less likely to use alcohol (2.0% vs 0.7%) and tobacco (12.4% vs 3.1%), less likely to have inadequate prenatal care during their pregnancies (8.3% vs 6.7%), and more likely to have inadequate weight gain (less than 22 pounds) (19.5% vs 22.6%). Infants born to foreign-born Filipino mothers had lower infant mortality rates (4.8 vs 5.8 per 1000 births) and were less likely to be low birthweight (6.1% vs 6.9%) than infants born to U.S.-born Filipino mothers.
**Alexander (19)**	1. Hawaii Linked Birth and Infant Death Data, 1979-87 2. 26,001 3. Cross-sectional study	Foreign-born Filipino mothers were more likely to have an infant born preterm (OR, 1.15; 95% CI, 1.05-1.26) and with a less favorable mean birth weight and gestational age than Hawaii-born Filipino mothers.
**Kieffer (20)**	1. U.S. birth certificate data. 1994-1996 2. 89,766 3. Cross-sectional study	Foreign-born Filipino mothers were significantly more likely to have gestational diabetes than U.S.-born Filipino mothers (OR, 1.27; 95% CI, 1.20-1.34). The adjusted odds ratio (age-only) was 1.11 (95% CI, 1.05-1.17) and adjusted odds ratio (age, prenatal care, education, marital status, parity) was 1.09 (95% CI, 1.03-1.16).
**Weitz (21)**	1. California birth certificate data,1989-1998, Family PACT^c^ client data, 1997-1998 2. Not available 3. Descriptive analysis	Among California's six largest API groups, Filipinos had the highest percentage of births among teenagers (6%). Filipino teens were also more likely than white teens to seek only a pregnancy test from a provider compared with whites (32% vs 22%).
**Schumacher (22)**	1. San Francisco, immigrant and refugee school-aged children, 1982-1985 2. 195 3. Semi-longitudinal study	All immigrant groups had a significant and upward trend in height. Except for Filipino boys, all groups had significant catch-up in weight.
**Baruffi (23)**	1. Hawaii Special Supplemental Nutrition Program for Women, Infants, and Children, 1997-1998 2. 3583 3. Cross-sectional study	Among children aged 1 year, Filipinos (30.2%) were the most likely to be underweight (defined as weight-for-age <10th percentile) compared with Asians (23.1%), blacks (11.9%), whites (12.6%), Hawaiians (15.7%), Hispanics (12.2%), Samoans (3.4%), and other ethnicities (17.9%). Among children aged 2 to 4 year, Filipinos (19.0%) were the shortest (defined as height-for-age < 10th percentile) compared with Asians (12.2%), blacks (6.2%), whites (9.0%), Hawaiians (13.6%), Hispanics (8.3%), Samoans (3.8%), and other ethnicities (10.1%). Compared with whites, Filipinos aged 2 to 4 years were more likely to be overweight, defined as BMI ≥95th percentile (OR, 1.76; 95% CI, 1.42-2.18)
**Greer (24)**	1. Hawaii oral health screening examinations, grades K-6, 1988-1989 2. 1280^d^ 3. Descriptive analysis	The percentage of Filipino children aged 5 years with baby bottle tooth decay (defined as caries in three or more of the four upper anterior primary teeth) was 32.19% compared with 4.14% for whites and second only to Southeast Asians at 33.33%. Filipino children aged 5 to 9 had mean 5.6 decayed filled teeth, the highest rate among all ethnic groups (the U.S. national average is 1.9); 45% of Filipino children aged 5 to 12 had unmet dental treatment needs compared with 21% of whites.
**Riedy (25)**	1. Saipan, Commonwealth of the Northern Mariana Islands, U.S., mothers with children <4 yo 2. 11^e^ 3. Focus groups	Low value of primary teeth and negative treatment experiences that parents have had with symptomatic dental care were major factors surrounding beliefs about oral health and behaviors. Even with these obstacles, mothers were open to new information and strategies to reduce the prevalence of early childhood caries.

a Number of Filipinos in study.

b Including Pacific Islanders.

c Planning, access, care, and treatment.

d Sample size only available for children aged 5 years; total sample of children aged 5 to 12 = 69,037.

e Denotes number of Filipino mothers.

**Table 2:** Studies of School-Aged Youths and Adolescents

**First Author**	**1. Data Source** **2. Sample Size^a^** **3. Methodology**	**Principal Findings**
**Harris (12)**	1. National Longitudinal Study of Adolescent Health, 1995 2. 673 3. Cross-sectional study	Adolescent health risk behaviors (i.e., ever having had sexual intercourse, young age at first intercourse, non-use of birth control at first intercourse, delinquency, violence, and use of controlled substances) increased with each generation for Filipino youths. Risky behaviors indexes show that foreign-born Filipino youth have significantly fewer health problems and engage in fewer risky behaviors than native white youths. For Filipino youth who were U.S.-born, the adverse effects of native birth and ethnic group on health status and risky behaviors diminished or became insignificant when family and neighborhood contexts were controlled.
**Beets (26)**	1. California public schools statewide mandated physical performance testing, 2002 2. 22,598 3. Cross-sectional study	Filipino males (35.8% vs 27.6%) and females (26% vs 24%) had higher prevalence of being overweight or of being at risk for overweight than whites. Filipino males were significantly more likely than white males to be overweight or at risk for overweight (OR, 1.66; 95% CI, 1.60-1.73). Filipino females were also significantly more likely than non-Hispanic white females to be at risk of being overweight or overweight (OR, 1.23; 95% CI, 1.17-1.28). Filipino males aged 10 and 11 years had slower mile run/walk times than non-Hispanic white males. All Filipino female age groups (aged 10-15 years) had slower mile run/walk times than non-Hispanic white females.
**Popkin (27)**	1. National Longitudinal Study of Adolescents,1996 2. 586 3. Cross-sectional study	Filipinos were less likely to be obese (18.5%) than whites (24.2%). Filipino females were less likely to be obese than Filipino males (12.8 vs 22.6%). Asian and Hispanic adolescents born in the United States were more than twice as likely to be obese as were first generation residents of the United States.
**Yates (28)**	1. Community college 2. 79 3. Cross-sectional study	Filipino males were the largest of all male groups and like Filipino females expressed a strong body- or self-dislike and preference for a smaller body.
**Robinson (29)**	1. 13 Northern California elementary schools 2. 70 3. Cross-sectional study	Filipino girls were less likely to have overweight concerns than African-Americans and Latinas but equally as likely as whites.
**Gordon-Larsen (30)**	1. National Longitudinal Study of Adolescent Health, 1996 2. Not available 3. Cross-sectional study	Filipinos were more likely to be inactive than whites (43.1% vs 28.0%). Being inactive was a significant risk factor for Filipinos (RR, 2.68; 95% CI, 1.74-4.14).
**California Department of Health Services (31)**	1. California Healthy Kids Survey, 2001-2003 2. 9943 3. Cross-sectional study	Filipinos had the highest lifetime asthma prevalence rate (23.3%) among API subgroups.
**Nelson (32)**	1. National tuberculosis surveillance system, 1993 to 2001 2. Not available 3. Cross-sectional study	Filipinos make up the second largest group of foreign-born children in the United States with tuberculosis.
**Pong (33)**	1. San Diego, school-based screenings, 1995 2. 676 3. Cross-sectional study	Filipinos (RR, 5; 95% CI, 2.1-11.8) were significantly more likely to have positive tuberculin skin test results than whites.
**Stevens (34)**	1. New York, San Francisco, and Los Angeles, study of passive-active prophylaxis for Asian hepatitis B carriers 2. 1478 3. Cross-sectional study	The prevalence of hepatitis B surface antigen was 5.1% among Philippine-born Asian women compared with 2.0% among U.S.-born Asian women.
**Jenkins (35)**	1. Los Angeles County, parents of fourth grade students, public elementary schools 2. 67 3. Cross-sectional study	Thirty-seven percent of Filipinos had all three of their required hepatitis B vaccinations. Nearly 13,000 Asian and Pacific Islander children living in the United States today will become infected with hepatitis B virus in the future, resulting in more than 600 liver carcinoma deaths.
**Grunbaum (36)**	1. Youth Risk Behavior Surveillance System, unpublished data collected by state and local education agencies, 1997 2. Not available 3. Cross-sectional study	Cigarette smoking among male students was 12% among Asians and 34% among Filipinos in San Diego, 13% among Chinese and 28% among Filipinos/Asians in San Francisco, and 26% among Hawaiians/part Hawaiians in Hawaii. The percentage of male students who had ever had sexual intercourse was 11% among Chinese and 28% among Filipinos/Asians in San Francisco, 29% among Asians and 49% among Filipinos in San Diego, and 34% among Hawaiians/part Hawaiians in Hawaii.
**Horan (37)**	San Francisco School District, CDC national surveillance survey 2. 152 3. Cross-sectional study	Filipino adolescents (32%) had sexual activity rates similar to those of white students (37%) and higher than Chinese students (13%).White students had higher HIV prevention scores than did Chinese and Filipino students, and whites had significantly greater ability to communicate with others about HIV disease and prevention. Chinese and Filipino students had fewer misconceptions about HIV than did white students.
**Chung (38)**	1. Los Angeles, various neighborhoods, 2001-2002 2. 85^b^ 3. Qualitative analysis with focus groups	Parent-child communication about sex, especially regarding values, was limited. Barriers to transmission of values were related to adolescent acculturation to the United States.
**Maxwell (39)**	1. Los Angeles County, face-to-face interviews, 1995 2. 211 3. Cross-sectional study	Higher self-efficacy and carrying condoms were the only variables that approached statistical significance in their relationship to condom use at last intercourse among Filipinos. Knowledge of HIV transmission, demographic variables, barriers to condom use, peer norms, and being comfortable asking a steady partner to routinely use condoms were not related to condom use at last intercourse.
**Wong (40)**	1. California Healthy Kids Survey and Hawaii Student Alcohol and Other Drug Use Survey (HSAD) 2. 8652 3. Cross-sectional study	Native Hawaiians and other Pacific Islanders tended to report the highest lifetime and 30-day rates of alcohol, tobacco, and other drug use, followed by whites, Filipinos, Japanese, and Chinese, suggesting an overall consistency in the patterns of use, irrespective of state of residence. Chinese respondents reported the lowest rates of use and need for treatment; in contrast, whites, Pacific Islanders, and Native Hawaiians reported the highest rates. Japanese and Filipinos fell in the middle.
**Chen (41)**	1. California Youth Tobacco Survey, 1990-1996 2. 595 3. Cross-sectional study	Among Asians, Filipinos have the highest lifetime smoking prevalence (18.9%). For all Asians, use of English at home and high English proficiency were associated with higher smoking prevalence (*P *= .021 and *P *= .040, respectively).
**Nemoto (42)**	1. San Francisco, California, drug users who were not currently enrolled in drug treatment programs 2. 31 3. Cross-sectional study and qualitative component	A majority of Chinese (66%) and Filipino (87%) participants reported using marijuana as a first illicit drug, whereas a large number of Vietnamese had begun by using cocaine (27%) or crack (31%). Filipino participants began using drugs at an earlier age (*M *= 14.3 years) than Chinese (*M* = 17.4 years) and Vietnamese (*M* = 21.7 years). Overall, immigrants (*M* = 18.9 years) began using drugs at a later age than U.S.-born participants (*M* = 15.0 years). Overall, Vietnamese women (*n* = 6) reported their initiation of drug use at a significantly later age (*M* = 27.8 years) than Chinese women (*M* = 15.2 years) and Filipino women (*M* = 15.5 years).
**Otsuki (43)**	1. California statewide survey of API and non-API American high school students, 1996 2. 1055 3. Cross-sectional study	Ninth grade Filipinos (male and female) had the highest proportion of alcohol (24.5%, 25.3%) and cigarette use (33.5%, 35.7%); 9th grade Filipino females had the highest marijuana use (10.7%); 12th grade Filipino students (male and female) had the highest proportion of cigarette use (40.8%, 42.8%) and marijuana use (18.6%, 17.1%). Among Filipino adolescent females in California, cigarette smoking is associated with depression (*P* = .01) and low self-esteem (*P* = .001). In contrast, alcohol use is associated with high self-esteem in Filipino males (*P* = .01).
**Hartz (44)**	1. Honolulu, Hawaii, 11th and 12th grade students and their parents 2. 14 3. Cross-sectional study	Polynesian parents exhibited more verbally and physically aggressive behavior toward their children than did white, Japanese, or Filipino parents. Filipino parents report having to alter their child-management style in the United States.
**Edman (45)**	1. Rural Hawaii, longitudinal survey investigating levels of psychopathological symptoms among adolescents 2. 285 3. Cross-sectional study	Compared with Filipino males, Filipino females were found to have higher Center for Epidemiologic Studies Depression Scale (CES-D) scores, with higher mean scores on the majority of the CES-D items. The few Filipino students who reported attempting suicide had moderately high to very high levels of reported depressive symptoms.
**Rumbaut (46)**	1. San Diego, California, Children of Immigrants Longitudinal Study, 1992 2. 818 3. Longitudinal study	Compared with Mexican, Cambodian, Lao, and other Asian and Latin American students, Filipinos and Vietnamese had statistically significant lower self-esteem scores.
**Wolf (47)**	1. Students at University of California, Davis, interviews with teachers, counselors, and principals in two high schools in Vallejo, California, 1995-1996 2. 22 3. Qualitative analysis with focus groups	Family offers a positive basis of Filipino identity for many children of immigrants but also is a deep source of stress and alienation, which has led to internal struggles and extreme despair as manifested by rates of depression and suicidal thoughts.

a Number of Filipinos in study.

b 41 parents and grandparents and 44 adolescents aged 14-18 years.

**Table 3:** Studies on Relevant Diseases in Filipino Adults

**First Author**	**1. Data Source** **2. Sample Size^a^** **3. Methodology**	**Principal Findings**
**Ryan (49)**	1. Northern California center treating coronary artery disease, 1992-1996 2. 527 3. Cross-sectional study	Filipinos had a higher incidence of hypertension (79% vs 61%) and diabetes (34.7% vs 24.1%) than whites. Hypercholesterolemia was similar in both groups. Obesity (18.3% vs 12.2%) and current smoking (21.5% vs 15.8%) were more common among whites than Filipinos. Age at presentation did not differ between groups. Following intervention in the catheterization lab (i.e., balloon angioplasty, rotational ablation, or stent placement), morbidity and mortality were higher among Filipinos than whites (4.2% vs 1.3%).
**Klatsky (50)**	1. Northern California Kaiser, 1978-1985 2. 4211 3. Cross-sectional study	Among Asians (Chinese, Japanese, other Asian), Filipino men and women had the highest prevalence of obesity and hypertension. Compared with Chinese men, Filipino men were more likely to be obese (OR, 1.9; 95% CI, 1.7-2.3) or hypertensive (OR, 1.3; 95% CI, 1-1.6). Compared with Chinese women, Filipino women were more likely to be obese (OR, 2.7; 95% CI, 2.3-3.2) or hypertensive (OR, 1.5; 95% CI, 1.2- 1.9). Birthplace in the United States was associated with increased risk of obesity among men of all ethnic subsets.
**Stavig (51)**	1. California Hypertension Survey, 1979 2. 422 3. Cross-sectional study	The prevalence of hypertension for Filipinos was 26.6%, second only to African Americans (33.8%). Prevalence among Filipino men aged 18 to 49 (30.5%) exceeded that of African Americans (28.3%) as did the rate for Filipino women aged 50 or older (65.2% vs 63.1%). Filipinos' rate of uncontrolled elevated blood pressure (24.5%) approached the well-documented high rate for African Americans (26.1%).
**Grandinetti (52)**	1. Rural Hawaii, 1997–2000 2. 197 3. Cross-sectional study	Despite significant differences in the prevalence of overweight and abdominal obesity, the prevalence of metabolic syndrome was similar in all ethnic groups other than white. Filipinos had the highest adjusted odds for prevalent metabolic syndrome (prevalence OR, 4.2; 95% CI, 2.4-7.3).
**Novotny (53)**	1. Honolulu, Hawaii, year not specified 2. 74 3. Cross-sectional study	Filipino women had higher subscapular (upper body) skin-fold thicknesses than did whites and had a greater percentage of body fat for the same value of BMI. Subscapular skin-fold thickness was the strongest correlate of diastolic blood pressure. No measures of body fat were associated with serum cholesterol.
**Araneta (54)**	1. San Diego, CA, Filipino comparison cohort to Rancho Bernardo Heart and Chronic Disease Study, 1992-1999 2. 294 3. Cross-sectional study	Filipino and white women did not differ in mean age, BMI, percentage of body fat, or waist-to-hip ratio. However, Filipino women had larger waist circumferences and higher percentages of truncal fat. Compared with whites, Filipino women were less likely to be obese (BMI ≥30 kg/m^2^) (8.8% vs 14%), to smoke, to consume alcohol, or to take postmenopausal estrogen; Filipino women also had lower levels of HDL cholesterol. Compared with whites, Filipino women had higher prevalence of type 2 diabetes by oral glucose tolerance test criteria (36% vs 9%) and metabolic syndrome (34% vs 13%). These differences persisted after adjusting for age, body size, fat distribution, percentage of body fat, smoking, alcohol consumption, exercise, and estrogen therapy.

a Number of Filipinos in study.

## References

[1] Stoddard JJ, Back MR, Brotherton SE (2000). The respective racial and ethnic diversity of US pediatricians and American children. Pediatrics.

[2] Brahan D, Bauchner H (2005). Changes in reporting of race/ethnicity, socioeconomic status, gender, and age over 10 years. Pediatrics.

[3] Surveys and data collection systems.

[4] (2002). U.S. Census Bureau. The Asian population: 2000.

[5] (2004). U.S. Census Bureau. We the people: Asians in the United States.

[6] Dela Cruz M, Agbayani-Siewert P, Lai E, Arguelles D (2003). Filipinos. The new face of Asian Pacific America: numbers, diversity, and change in the 21st century.

[7] (2001). U.S. Census Bureau. Census 2000 summary file 2 (SF2): 100-percent data files.

[8] Dela Cruz FA, McBride MR, Compas LB, Calixto PR, Van Derveer CP (2002). White paper on the health status of Filipino Americans and recommendations for research. Nurs Outlook.

[9] Gomez SL, Kelsey JL, Glaser SL, Lee MM, Sidney S (2004). Immigration and acculturation in relation to health and health-related risk factors among specific Asian subgroups in a health maintenance organization. Am J Public Health.

[10] (1999). U.S. Department of Health and Human Services. Healthy People 2010: national health promotion and disease prevention objectives.

[11] Yu SM, Huang ZJ, Singh GK (2004). Health status and health services utilization among US Chinese, Asian Indian, Filipino, and other Asian/Pacific Islander children. Pediatrics.

[12] Harris K, Hernandez DJ (1998). The health status and risk behavior of adolescents in immigrant families. Children of immigrants: health, adjustment, and public assistance.

[13] Mathews TJ, Menacker F, MacDorman MF (2004). Infant mortality statistics from the 2002 period: linked birth/infant death data set. Natl Vital Stat Rep.

[14] Fuentes-Afflick E, Hessol NA (1997). Impact of Asian ethnicity and national origin on infant birth weight. Am J Epidemiol.

[15] Alexander GR, Baruffi G, Mor JM, Kieffer EC, Hulsey TC (1993). Multiethnic variations in the pregnancy outcomes of military dependents. Am J Public Health.

[16] Walton DL, Norem CT, Schoen EJ, Ray GT, Colby CJ (1999). Second-trimester serum chorionic gonadotropin concentrations and complications and outcome of pregnancy. N Engl J Med.

[17] Martin JA, Hamilton BE, Sutton PD, Ventura SJ, Menacker F, Munson ML (2003). Births: final data for 2002. Natl Vital Stat Rep.

[18] Landale NS, Oropesa RS, Gorman BK, Hernandez DJ (1998). Immigration and infant health: birth outcomes of immigrant and native-born women. Committee on the Health and Adjustment of Immigrant Children and Families. Children of immigrants: health, adjustment, and public assistance.

[19] Alexander GR, Baruffi G, Mor J, Kieffer E (1992). Maternal nativity status and pregnancy outcome among U.S.-born Filipinos. Soc Biol.

[20] Kieffer EC, Martin JA, Herman WH (1999). Impact of maternal nativity on the prevalence of diabetes during pregnancy among U.S. ethnic groups. Diabetes Care.

[21] Weitz T, Harper C, Mohllajee A (2001). Teen pregnancy among Asians and Pacific Islanders in California: final report.

[22] Schumacher LB, Pawson IG, Kretchmer N (1987). Growth of immigrant children in the newcomer schools of San Francisco. Pediatrics.

[23] Baruffi G, Hardy CJ, Waslien CI, Uyehara SJ, Krupitsky D (2004). Ethnic differences in the prevalence of overweight among young children in Hawaii. J Am Diet Assoc.

[24] Greer M, Tengan S (1998). Dental caries in early childhood among Native Hawaiians. Pacific Health Dialog.

[25] Riedy CA, Weinstein P, Milgrom P, Bruss M (2001). An ethnographic study for understanding children's oral health in a multicultural community. Int Dent J.

[26] Beets MW, Pitetti KH (2004). One-mile run/walk and body mass index of an ethnically diverse sample of youth. Med Sci Sports Exerc.

[27] Popkin BM, Udry JR (1998). Adolescent obesity increases significantly in second and third generation U.S. immigrants: the National Longitudinal Study of Adolescent Health. J Nutr.

[28] Yates A, Edman J, Aruguete M (2004). Ethnic differences in BMI and body/self-dissatisfaction among Whites, Asian subgroups, Pacific Islanders, and African-Americans. J Adolesc Health.

[29] Robinson TN, Chang JY, Haydel KF, Killen JD (2001). Overweight concerns and body dissatisfaction among third-grade children: the impacts of ethnicity and socioeconomic status. J Pediatr.

[30] Gordon-Larsen P, McMurray R, Popkin B (1999). Adolescent physical activity and inactivity vary by ethnicity: The National Longitudinal Study of Adolescent Health. J Pediatr.

[31] (2004). California Department of Health Services. Asthma in Schools: Results from the California Healthy Kids Survey, 2001-2003. California Asthma Facts.

[32] Nelson LJ, Schneider E, Wells CD, Moore M (2004). Epidemiology of childhood tuberculosis in the United States, 1993-2001: the need for continued vigilance. Pediatrics.

[33] Pong AL, Anders BJ, Moser KS, Starkey M, Gassmann A, Besser RE (1998). Tuberculosis screening at 2 San Diego high schools with high-risk populations. Arch Pediatr Adolesc Med.

[34] Stevens CE, Toy PT, Tong MJ, Taylor PE, Vyas GN, Nair PV (1985). Perinatal hepatitis B virus transmission in the United States. Prevention by passive-active immunization. JAMA.

[35] Jenkins CN, Buu C, Berger W, Son DT (2001). Liver carcinoma prevention among Asian Pacific Islanders. Getting hepatitis B shots into arms. Cancer.

[36] Grunbaum JA, Lowry R, Kann L, Pateman B (2000). Prevalence of health risk behaviors among Asian American/Pacific Islander high school students. J Adolesc Health.

[37] Horan PF, DiClemente RJ (1993). HIV knowledge, communication, and risk behaviors among white, Chinese-, and Filipino-American adolescents in a high-prevalence AIDS epicenter: a comparative analysis. Ethn Dis.

[38] Chung PJ, Borneo H, Kilpatrick SD, Lopez DM, Travis R, Lui C (2005). Parent-adolescent communication about sex in Filipino American families: a demonstration of community-based participatory research. Ambul Pediatr.

[39] Maxwell AE, Bastani R, Warda US (2000). Knowledge and attitudes toward condom use — do they predict behavior among Filipino Americans?. Ethn Dis.

[40] Wong MM, Klingle RS, Price RK (2004). Alcohol, tobacco, and other drug use among Asian American and Pacific Islander Adolescents in California and Hawaii. Addict Behav.

[41] Chen X, Unger JB, Cruz TB, Johnson CA (1999). Smoking patterns of Asian-American youth in California and their relationship with acculturation. J Adolesc Health.

[42] Nemoto T, Aoki B, Huang K, Morris A, Nguyen H, Wong W (1999). Drug use behaviors among Asian drug users in San Francisco. Addict Behav.

[43] Otsuki TA (2003). Substance use, self-esteem, and depression among Asian American adolescents. J Drug Educ.

[44] Hartz DT (1995). Comparative conflict resolution patterns among parent-teen dyads of four ethnic groups in Hawaii. Child Abuse Negl.

[45] Edman JL, Andrade NN, Glipa J, Foster J, Danko GP, Yates A (1998). Depressive symptoms among Filipino American adolescents. Cult Divers Ment Health.

[46] Rumbaudt RG, Portes A (1996). The crucible within: ethnic identity, self-esteem, and segmented assimilation among children of immigrants. The new second generation.

[47] Wolf D (1997). Family secrets: transnational struggles among children of Filipino immigrants. Sociol Perspect.

[48] Sloan NR (1963). Ethnic distribution of diabetes mellitus in Hawaii. JAMA.

[49] Ryan C, Shaw R, Pliam M, Zapolanski AJ, Murphy M, Valle HV (2000). Coronary heart disease in Filipino and Filipino-American patients: prevalence of risk factors and outcomes of treatment. J Invasive Cardiol.

[50] Klatsky AL, Armstrong MA (1991). Cardiovascular risk factors among Asian Americans living in northern California. Am J Public Health.

[51] Stavig GR, Igra A, Leonard AR (1988). Hypertension and related health issues among Asians and Pacific Islanders in California. Public Health Rep.

[52] Grandinetti A, Chang HK, Theriault A, Mor J (2005). Metabolic syndrome in a multiethnic population in rural Hawaii. Ethn Dis.

[53] Novotny R, Davis J, Ross P, Wasnich R (1998). Adiposity and blood pressure in a multiethnic population of women in Hawaii. Ethn Health.

[54] Araneta MR, Wingard DL, Barrett-Connor E (2002). Type 2 diabetes and metabolic syndrome in Filipina-American women : A high-risk nonobese population. Diabetes Care.

[55] Halfon N, Hochstein M (2002). Life course health development: an integrated framework for developing health, policy, and research. Milbank Q.

[56] Wise P (2004). The transformation of child health in the United States. Health Aff (Millwood).

[57] Anderson JN (1983). Health and illness in Pilipino immigrants. West J Med.

[58] Fuligni AJ (1998). Authority, autonomy, and parent-adolescent conflict and cohesion: a study of adolescents from Mexican, Chinese, Filipino, and European backgrounds. Dev Psychol.

[59] Javier JR, Chamberlain L, Huffman L, Mendoza F (2006). Filipino American families and intergenerational communication about sex. Ambul Pediatr.

[60] Richardson LP, DiGiuseppe D, Garrison M, Christakis DA (2003). Depression in Medicaid-covered youth: differences by race and ethnicity. Arch Pediatr Adolesc Med.

[61] Bui KV, Takeuchi DT (1992). Ethnic minority adolescents and the use of community mental health care services. Am J Community Psychol.

[62] Yamada S, Caballero J, Matsunaga DS, Agustin G, Magana M (1999). Attitudes regarding tuberculosis in immigrants from the Philippines to the United States. Fam Med.

[63] Tompar-Tiu A, Sustento-Seneriches J (1995). Depression and other mental health issues: the Filipino American experience.

[64] Ghosh C (2003). Healthy People 2010 and Asian Americans/Pacific Islanders: defining a baseline of information. Am J Public Health.

[65] Dela Cruz FA, Padilla GV, Agustin EO (2000). Adapting a measure of acculturation for cross-cultural research. J Transcult Nurs.

[66] Hishinuma ES, Miyamoto RH, Nishimura ST, Nahulu LB, Andrade NN, Makini GK (2000). Psychometric properties of the state-trait anxiety inventory for Asian/Pacific-islander adolescents. Assessment.

[67] Edman JL, Danko GP, Andrade N, McArdle JJ, Foster J, Glipa J (1999). Factor structure of the CES-D (Center for Epidemiologic Studies Depression Scale) among Filipino-American adolescents. Soc Psychiatry Psychiatr Epidemiol.

[68] Srinivasan S, Guillermo T (2000). Toward improved health: disaggregating Asian American and Native Hawaiian/Pacific Islander data. Am J Public Health.

[69] (2003). Cardiovascular risk in the Filipino community, formative research from Daly City and San Francisco, California.

[70] Minkler M, Wallerstein N (2003). Community-based participatory research for health.

[71] Computer Retrieval of Information on Scientific Projects (CRISP) Database.

[72] Huang L, Lee Y, Arganza G (2004). Promising approaches in youth development and risk prevention for Asian American/Pacific Islander youth: voices from the field.

[73] Yancey AK, Siegel JM, McDaniel KL (2002). Role models, ethnic identity, and health-risk behaviors in urban adolescents. Arch Pediatr Adolesc Med.

[74] Coll CG, Szalacha LA (2004). The multiple contexts of middle childhood. The Future of Children: Children of Immigrant Families.

[75] Brown ER, Holtby S, Zahnd E, Abbott GB (2005). Community-based participatory research in the California Health Interview Survey. Prev Chronic Dis.

[76] Tervalon M, Murray-Garcia J (1998). Cultural humility versus cultural competence: a critical distinction in defining physician training outcomes in multicultural education. J Health Care Poor Underserved.

[77] (1999). Culturally effective pediatric care: education and training issues. American Academy of Pediatrics Committee on Pediatric Workforce. Pediatrics.

